# POEMS syndrome with undetectable M-protein: a case report and literature review

**DOI:** 10.1186/s13000-024-01502-4

**Published:** 2024-06-07

**Authors:** Han-Yue Xue, Lin Zhou, Qin-Zhao Yuan, Yang Zhang, Yi-Qun Hao, Shao-Wei Chen, Hong-Kun Wang, Fang Wei

**Affiliations:** 1 The First Clinical Medical College of Shanxi Medical University, 56 Xinjian South Road, Yingze District, Taiyuan, Shanxi China; 2 Department of Nephropathy, Bao Ji High-Tech Hospital, Bao Ji, Shaanxi People’s Republic of China; 3 Department of Hematology, The First Hospital of Shanxi Medical University, 85 Jiefang South Road, Yingze District, Taiyuan, Shanxi People’s Republic of China; 4 Department of Bone and Soft Tissue Oncology, Cancer Hospital of the Chinese Academy of Medical Sciences, Shanxi Hospital, Shanxi Provincial Cancer Hospital, Taiyuan, Shanxi People’s Republic of China; 5 Department of Rheumatology and Immunology, The First Hospital of Shanxi Medical University, Taiyuan, Shanxi People’s Republic of China; 6 Department of Pathology, The First Hospital of Shanxi Medical University, Taiyuan, Shanxi People’s Republic of China

**Keywords:** POEMS syndrome, M-protein, Plasma cell, Diagnosis

## Abstract

**Background:**

Polyneuropathy, organomegaly, endocrinopathy, M-protein, and skin changes (POEMS) syndrome is a rare plasma cell (PC) neoplasm with associated paraneoplastic syndrome. According to the current diagnostic criteria, peripheral polyneuropathy and monoclonal PC proliferative disorder represent two mandatory criteria.

**Case presentation:**

We report a 54-year-old male with peripheral neuropathy of bilateral lower limbs, sclerotic bone lesions, elevated vascular endothelial growth factor (VEGF) levels, splenomegaly, extravascular volume overload, endocrinopathy, and skin hemangiomas. Of note, serum and urine protein electrophoresis (PEP) and immunofixation electrophoresis (IFE) of this patient indicated undetectable M-protein and the normal ratio of free light chains κ and λ (FLC-R (κ/λ)). No monoclonal PCs were found in bone marrow examinations or biopsy of diseased bones. However, his clinical manifestations matched most of the diagnostic criteria. After excluding other diseases that are easily confused with POEMS syndrome, the diagnosis of variant POEMS syndrome with undetectable M-protein was proposed. The patient obtained clinically significant improvement and elevated VEGF returned to normal after 6 months of treatment with lenalidomide plus dexamethasone.

**Conclusions:**

Monoclonal PC dyscrasia (M-protein) while being a mandatory criterion for POEMS syndrome is undetectable in a considerable amount of patients that otherwise demonstrate typical symptoms. Here, we reported a case of variant POEMS syndrome with featured clinical manifestations, elevated VEGF levels, and good response to therapies targeting PCs but no evidence of M-protein. Therefore, negative results in M-protein and monoclonal PCs aren’t enough to reject the diagnosis of POEMS syndrome. It is imperative to recognize the variant form of POEMS syndrome.

## Background

POEMS syndrome is a paraneoplastic syndrome associated with the malignant clonal proliferation of PCs, which is also recognized as osteosclerotic myeloma, Crow Fukase syndrome, and Takatsuki disease [1–3]. Although the main clinical manifestations have been characterized, the precise mechanisms explaining the development, progression, and clinical manifestations of this disease remain elusive. Most studies believe that cytokines generated by monoclonal PCs, such as interleukin-6 (IL-6), interleukin-1β (IL-1β), and tumor necrosis factor-α (TNF-α), play indispensable roles in POEMS syndrome by interacting with M-protein and/or VEGF [4].

According to the latest diagnostic criteria of POEMS syndrome revised by The International Myeloma Working Group in 2017, two mandatory and at least one major and minor criteria are required for the diagnosis of POEMS syndrome. Mandatory criteria include peripheral polyneuropathy and monoclonal PC proliferative disorder [3]. Major criteria consist of Castleman disease, osteosclerotic lesions, and elevated serum or plasma VEGF level. Minor criteria include organomegaly (hepatomegaly, splenomegaly, or lymphadenopathy), extravascular volume overload (peripheral edema, ascites, or pleural effusions), endocrinopathy (adrenal, pituitary, gonadal, parathyroid, thyroid, parathyroid, or pancreatic), skin changes (hyperpigmentation, hypertrichosis, glomeruloid hemangioma, plethora, acrocyanosis, flushing, or white nails), papilledema, and thrombocytosis [5]. M-protein produced by monoclonal PCs provides a fundamental element for diagnosing POEMS syndrome. As in most reports, the majority of patients exhibited positive M-protein [1, 2, 6, 7]. Here, we present a case of POEMS syndrome with undetectable M-protein to strengthen the understanding of it, so that patients with analogous symptoms in the future can be detected, diagnosed, and treated early.

## Case presentation

### Course of disease

A 54-year-old male presented with numbness and edema in bilateral lower limbs in 2018 and then underwent positron emission tomography-computed tomography (PET-CT) at a hospital, indicating intense metabolic activity in multiple bones throughout the body. However, a definitive diagnosis wasn’t made. Subsequently, he experienced progressive numbness and edema as well as developed weakness in bilateral lower limbs for which he intermittently received Chinese medicine. Unfortunately, symptoms didn’t improve. In Sep 2022, he underwent a chest CT at another hospital, showing bilateral mild pleural effusion. For further diagnosis and treatment, he was admitted to the Rheumatology and Immunology Department of our hospital in Oct 2022. Since the disease’s onset, he has been frustrated and lethargic and has a normal diet and sleep, regular voiding, and no significant weight changes. Meanwhile, he denied a history of chronic diseases, tobacco and alcohol use, food and drug allergies, and genetic disorders.

### Physical examination

**Fig. 1:**
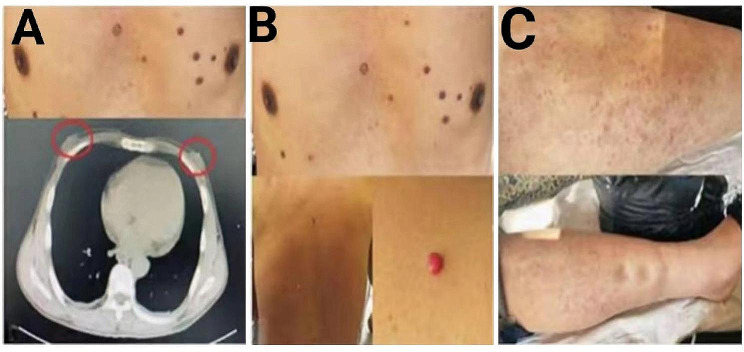
The results of the patient’s physical examination. (A) Breast protrusion on both sides could be seen through visual examination and CT; (B) Hemangiomas could be seen in the anterior chest and back; (C) The patient had concave edema in bilateral lower limbs. Abbreviations: CT: Computed Tomography

The patient’s vital signs were stable. He had no signs of jaundice, anemia, or cyanosis. His bilateral breasts were significantly protruded (Fig. 1A). Besides, fresh red hemangiomas were scattered throughout the anterior portion of his chest and back, with a maximum size of about 0.40 cm×0.50 cm (Fig. 1B). There were no apparent abnormalities in the cardiopulmonary and abdominal examination. However, pitting edema was noted in bilateral lower limbs (Fig. 1C). Neurological examination revealed normal cranial nerves and no cerebellar signs. However, there was diminished power in the bilateral lower limbs (4/5) by the Manual Muscle Test (MMT). Physiological reflexes were present and pathological reflexes were negative. Deep and shallow sensations were weakened below the bilateral ankle joints. The upper limb examination was unremarkable.

### Auxiliary examinations

#### Laboratory examinations

**Fig. 2:**
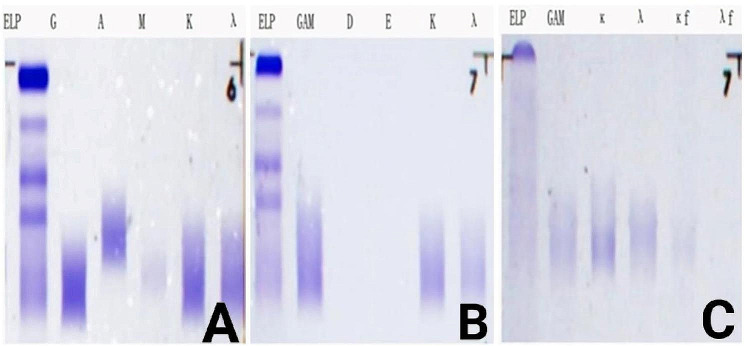
The results of the patient’s serum IFE and urine BJP electrophoresis. (A) No abnormal monoclonal bands were found in IgA, IgG, IgM, κ and λ swimming lanes in serum IFE; (B) No abnormal monoclonal bands were found in IgD, IgE, κ and λ swimming lanes in serum IFE; (C) No abnormal monoclonal bands were found in swimming lanes in urine BJP electrophoresis and BJP was negative. Abbreviations: IFE: immunofixation electrophoresis; BJP: Bence-Jones protein; ELP: electrophoresis; GAM: IgG, IgA, and IgM; G: IgG; A: IgA; M: IgM; D: IgD; E: IgE; κ: κ chain; λ: λ chain; κf: free κ chain; λf: free λ chain

His complete blood count, clotting test, and blood glucose were generally normal. IgA, IgG, IgM, and IgE were also within the normal range. His serum/urine PEP and IFE showed negative M-protein. The levels of serum-free light chains κ and λ were increased, while the ratio of κ/λ in serum was normal (Fig. 2A and C). The levels of serum VEGF were elevated. FT3 and FT4 were decreased, and TSH was increased. Sex hormone measurement revealed increased prolactin (PRL), follicular estrogen (FSH), estradiol (E2), and progestational hormone (P) but normal testosterone (T). ACTH was increased and cortisol (COR) at 8:00 AM was decreased. Rheumatism-related indicators, hepatitis B and C, syphilis, and AIDS serology tests were all negative. The detailed results are shown in Table 1.

**Table 1:** The results of laboratory examinations on admission

Blood routine		DBIL	4.10 µmol/L	Immunological tests		Endocrine tests	
WBC	5.10*10^9 /L	IBIL	13.70 µmol/L	IgA	3.76 g/L	**TSH**	**8.66 µIU/ml**
RBC	4.42*10^12 /L	**Renal function**		IgG	10.02 g/L	Ref 0.27–4.20 µIU/ml	
HGB	139 g/L	Urea	5.41 mmol/L	IgM	1.06 g/L	**FT3**	**2.80 pmol/L**
PLT	283*10^9 /L	CRE	65 µmol/L	IgE	17 KU/L	Ref 3.10–6.80 pmol/L	
NEUT %	60.70%	UA	313 µmol/L	C3	0.92 g/L	**FT4**	**9.96 pmol/L**
LYMPH %	28.20%	**Electrolyte tests**		C4	0.19 g/L	Ref 12–22 pmol/L	
**MONO %**	**10.20%**	Na^+^	140 mmol/L	**24hUTP**	**354 mg**	**PRL**	**500 mIU/ml**
**Blood biochemistry**		K^+^	3.91 mmol/L	**sFLC-κ**	**79.30 mg/L**	Ref 56–278 mIU/ml	
**β2-MG**	**4.79 mg/L**	Cl^−^	106.10 mmol/L	Ref 3.30–19.40 mg/L		**FSH**	**19.30 mIU/ml**
Ref 0.80–2.40 mg/L		Mg	0.78 mmol/L	**sFLC-λ**	**80.09 mg/L**	**LH**	**14.78 mIU/ml**
LDH	132 U/L	**Ca**	**1.92 mmol/L**	Ref 5.71–26.30 mg/L		**E2**	**161 pmol/L**
GLU	4.75 mmol/L	Ref 2.11–2.52 mmol/L		**uFLC-κ**	**184 mg/L**	**P**	**0.33 nmol/L**
**Liver function**		**P**	**1.73 mmol/L**	Ref 0.39–15.10 mg/L		T	7.50 nmol/L
ALT	33 U/L	Ref 0.85–1.51 mmol/L		**uFLC-λ**	**50.90 mg/L**	**ACTH**	**16.52 pmol/L**
AST	21 U/L	**Blood lipids**		Ref 0.81–10.10 mg/L		COR 0:00 AM	117 nmol/L
**TP**	**58.20 g/L**	**TC**	**2.64 mmol/L**	sFLC-R (κ/λ)	0.99	**COR8:00 AM**	**149.50 nmol/L**
**ALB**	**31.50 g/L**	**TG**	**1.72 mmol/L**	uFLC-R (κ/λ)	3.61	COR 4:00 PM	212.80 nmol/L
GLB	26.70 g/L	**HDL-C**	**0.55 mmol/L**	**ESR**	**40 mm/h**	**VEGF**	**254.61 pg/ml**
TBIL	17.80 µmol/L	LDL-C	1.58 mmol/L	**PCT**	**1.22 ng/ml**	Ref <160 pg/ml	

All abnormal results have been bolded. Abbreviations: Ref: reference range; WBC: white blood cell; RBC: red blood cell; HGB: hemoglobin; PLT: platelet; NEUT: neutrophils; LYMPH: lymphocyte; MONO: monocyte; β2-MG: beta2-microglobulin; LDH: lactic dehydrogenase; GLU: glucose; ALT: alanine transaminase; AST: aspartate transaminase; TP: total protein; ALB: albumin; GLB: globulin; TBIL: total bilirubin; DBIL: direct bilirubin; IBIL: indirect bilirubin; CRE: creatinine; UA: uric acid; TC: total cholesterol; TG: total glyceride; HDL-C: High-density lipoprotein-cholesterol; LDL-C: Low-density lipoprotein-cholesterol; Ig: immunoglulin; C3:complement 3; C4: complement 4; UTP: urine total protein; sFLC: serum free light chain; uFLC: urine free light chain; FLC-R: ratio of free light chain; ESR: erythrocyte sedimentation rate; PCT: procalcitonin; TSH: thyroid stimulating hormone; FT3: free triiodothyronine; FT4: free tetraiodothyronine; PRL: prolactin; FSH: follicular estrogen; LH: luteinizing hormone; E2: estradiol; P: The former in electrolyte tests refers to inorganic phosphorus and the latter in endocrine tests refers to progestational hormone. ACTH: adrenocorticotropic hormone; T: testosterone; COR: cortisol; AM: ante meridiem; PM: post meridiem; VEGF: vascular endothelial growth factor

#### Imaging examinations

**Fig. 3:**
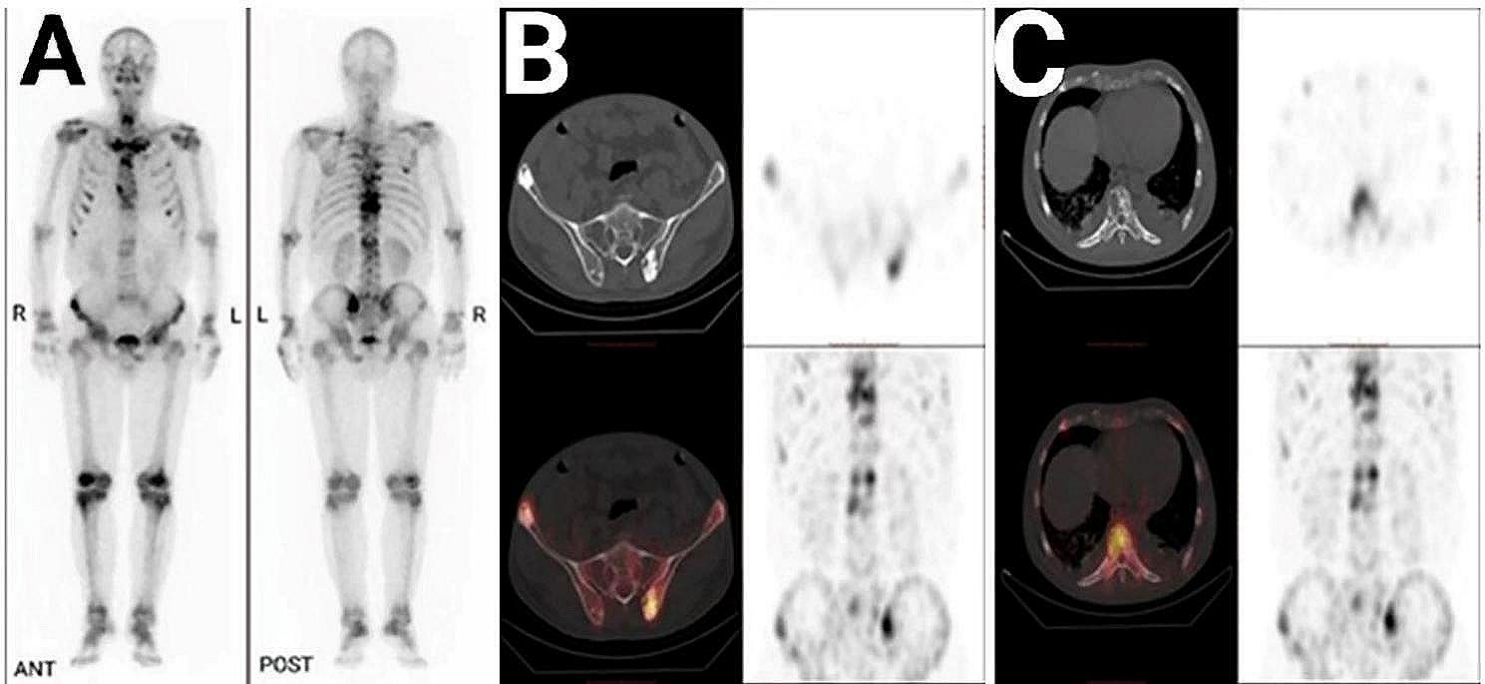
The results of bone scintigraphy with [99mTc]-MDP SPECT/CT. (A) Three hours after intravenous injection of the imaging agent, bone scintigraphy revealed enhanced bone metabolism in multiple parts of the body. The local SPECT/CT fusion images showed the coexistence of osteogenic and osteolytic bone destruction of (B) bilateral iliac crest, and (C) vertebrae and ribs. Abbreviations: MDP SPECT/CT: methylene-diphosphonate Single Photon Emission Computed Tomography/Computed Tomography; R: right; L: left; ANT: anterior; POST: posterior

The thyroid ultrasound showed two small hypoechoic nodes in the lower pole of the right lobe of the thyroid gland, the larger being 0.47 cm×0.29 cm. Echocardiography revealed bilateral atrial enlargement and pericardial effusion (small amount). Abdominal ultrasound displayed splenomegaly with an intercostal thickness of about 5.70 cm and a length of 16.30 cm. Chest and abdominal CT revealed enlarged lymph nodes in the bilateral axilla and mediastinum (the largest with a diameter of approximately 1.10 cm), as well as small amounts of pleural effusion and pericardial effusion. Bone scintigraphy with [99mTc]-methylene-diphosphonate (MDP) single-photon emission computed tomography/computed tomography (SPECT/CT) exhibited a symmetrical bilateral skeleton and mixed sclerotic and lytic bone lesions in bilateral ilium, vertebral bodies, and ribs. (Figure 3A and C).

#### Bone marrow (BM) examinations

**Fig. 4:**
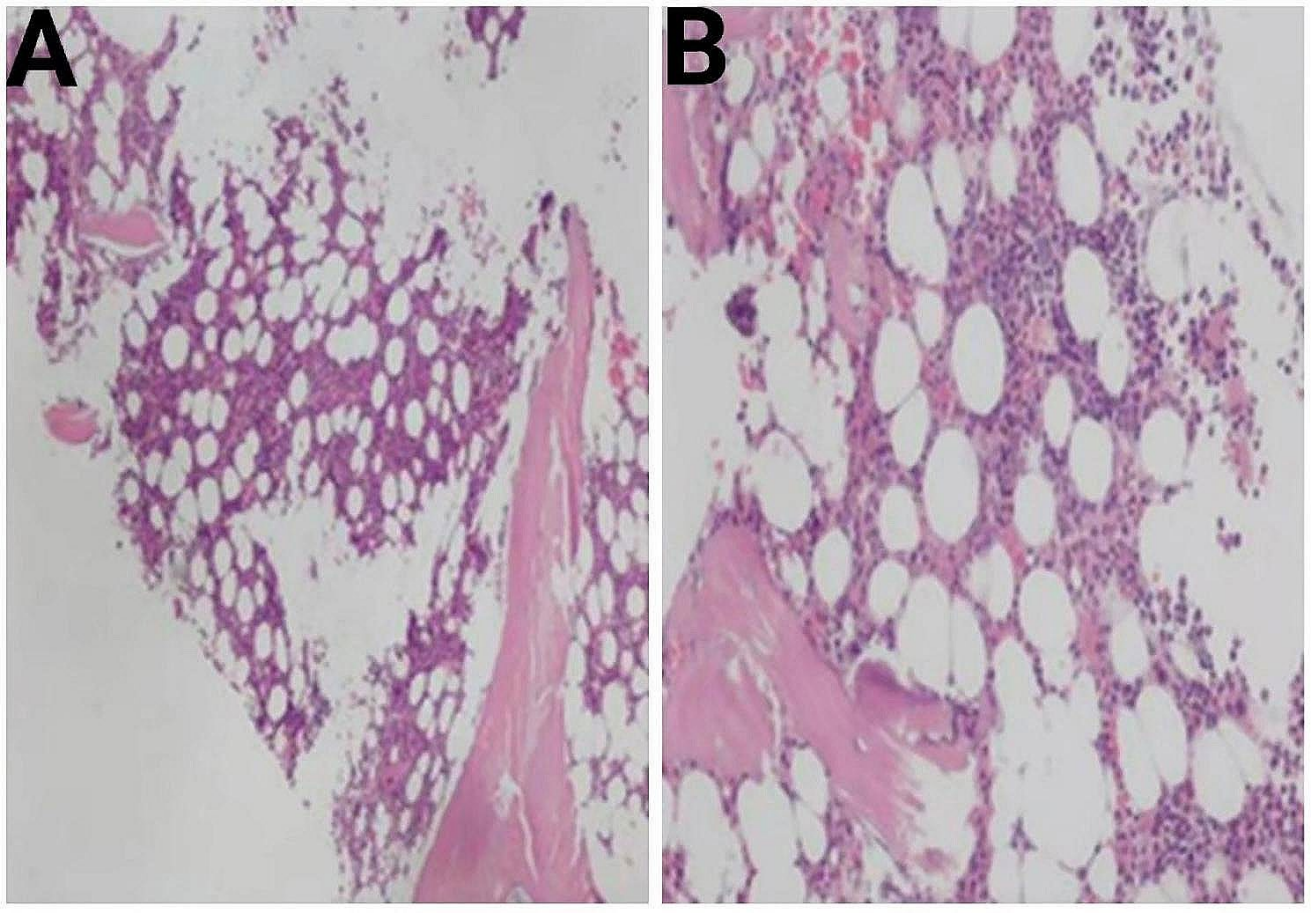
The results of BM biopsy. We selected one piece of gray-yellow tissue with a volume of 1.50 cm×0.20 cm×0.20 cm. Under the microscope, (A) (original magnification×40) and (B) (original magnification×100) showed that there was no increase in immature cells and PCs. Reticular fiber staining was scored MF-1. Abbreviations: BM: bone marrow; cm: centimeter; PC: plasma cell; MF: myelofibrosis

BM smears revealed that the proportion and morphology of red blood cells, granulocytes, and megakaryocytes were generally normal, and mature PCs constituted 2% of cells. Flow cytometry of BM specimens showed no significant evidence of monoclonal PCs and other abnormal cells (immunophenotypic abnormalities). Besides, the karyotype revealed normal male chromosomes. BM biopsy of the posterior superior iliac spine displayed no increase in the abundance of immature cells, lymphocytes, and PCs (Fig. 4A and B).

#### Bone biopsy

**Fig. 5:**
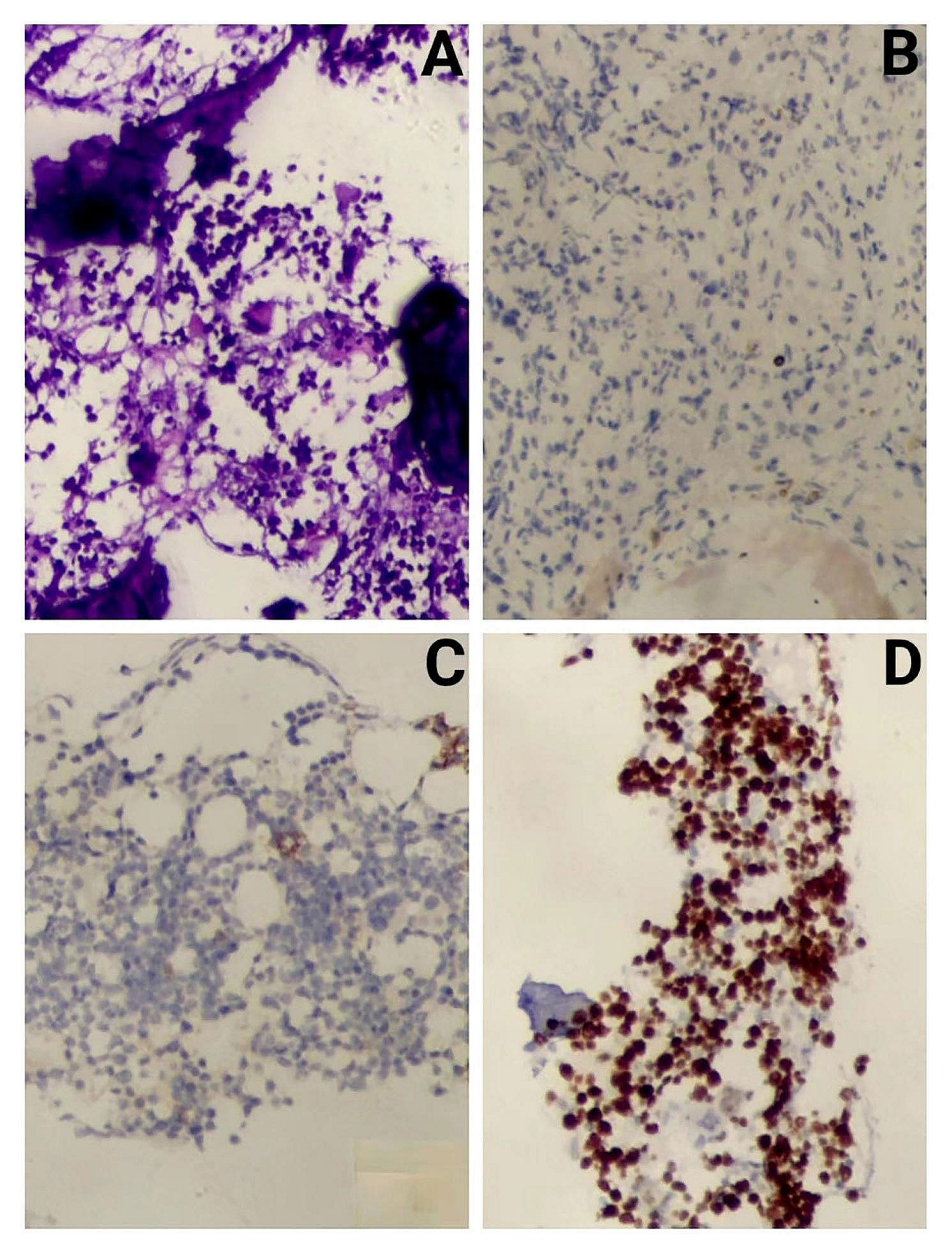
The results of bone biopsy (Envision 10 × 10). A CT-guided biopsy of the posterior superior iliac spine was performed. (A) was the H&E stained image, which showed no significantly abnormal distribution of hematopoietic tissues, with a few mineralized bone tissues and BM. The immunohistochemical results revealed (B) Langerin (-), (C) CD138 (-), and (D) Ki67 (80% +). Abbreviations: CT: Computed Tomography; H&E: hematoxylin-eosin; BM: bone marrow

He experienced a CT-guided coarse needle puncture of the posterior superior iliac spine. Pathological assessments indicated no increase in the number of PCs and lymphocytes. The results of immunohistochemistry indicated CK (-), LCA (+), langerin (-), CD68 (+), CD163 (+), CD1α (-), S100 (-), Ki-67 (80% +), CD138 (-), and CD56 (-) (Fig. 5A and D).

### Differential diagnosis

#### Multiple myeloma (MM)

The classic symptoms of MM are elevated calcium level, renal insufficiency, anemia, and lytic bone lesions, which were not found in our case. MM doesn’t generally present with peripheral neuropathy, organ enlargement, multiple endocrine gland abnormalities, skin-specific changes, sclerotic bone lesions, and increased VEGF levels, which all were present in our patient. In addition, the proportion of monoclonal PCs in the BM of MM should be at least 10%, while monoclonal PCs in the BM of our patient were not present.

#### Castleman disease (CD)

The CD is clinically divided into focal and multicenter types. The former is more common in young people. Multicentric Castleman disease (MCD) also presents with lymphadenopathy, hepatomegaly, splenomegaly, rash, and cavity effusion. However, it is usually not accompanied by peripheral neuropathy, sclerotic bone lesions, endocrinopathy, and increased VEGF levels, which were present in our patient. Patients with MCD are prone to infection and lymphoma. MCD usually has an aggressive course and poor overall survival (OS). However, the onset of this patient is insidious, and the course is chronic and stable, just like POEMS syndrome.

#### Guillain barre syndrome (GBS)

Both GBS and POEMS syndrome can lead to motor neuron paralysis, but GBS is not accompanied by visceral enlargement, endocrinopathy, skin changes, bone lesions, and increased VEGF, which all were present in our patient.

#### Chronic inflammatory demyelinating polyradiculoneuropathy (CIDP)

POEMS syndrome and CIDP affect the motor and sensory nerves, slowing nerve conduction. However, CIDP doesn’t present with organ enlargement, endocrinopathy, M-protein deposition, skin changes, bone lesions, or increased VEGF levels, most of which were present in our patient.

### Final diagnosis

Although the M-protein of the patient was undetectable, he had peripheral neuropathies in bilateral lower limbs. Additionally, he showed signs of male breast development and characteristic hemangiomas in the anterior chest and back. Hormonal tests revealed hypothyroidism, hyperprolactinemia, hypoadrenalism, and feminization. More importantly, he had an increased VEGF level, and SPECT/CT revealed sclerotic bone lesions. Ultrasound and CT showed enlargement of lymph nodes, splenomegaly, and extravascular volume overload. After excluding the disorders that are easily confused with POEMS syndrome, we proposed the diagnosis of POEMS syndrome with undetectable M-protein.

### Treatment and follow-up

Then the patient was treated with the RD regimen (25 mg/d of lenalidomide capsules on days 1–21, 20 mg/d of dexamethasone tablets on days 1–4 and 8–11, each cycle is 28 days). At the same time, he received levothyroxine sodium tablets (25 µg/d) to improve hypothyroidism and aspirin to prevent coagulation. Nearly 6 months after receiving the treatment, the numbness and edema of bilateral lower limbs significantly improved, and the color of skin hemangiomas altered from fresh red to dark red. Elevate serum VEGF and free light chains have returned to normal. All indicators were within the normal range on the thyroid, adrenal gland, and sex hormone tests. Imaging examinations exhibited normal volume of lymph nodes, normal size and morphology of the liver and spleen, and no signs of extravascular volume overload. Bone X-rays still indicated high-density shadows in multiple bone areas throughout the body. Thus, we will continue the follow-up.

## Discussion

We report a case with clinical manifestations highly similar to those of POEMS syndrome. Surprisingly, no demonstrable monoclonal gammopathies or monoclonal PCs were detected by existing detection methods. Kastritis E et al. reported that reduced VEGF levels can parallel improve clinical symptoms and restore normal bone metabolism [8]. Thus, we evaluated all of his clinical features and changes in relevant indicators including serum VEGF levels before and after treatment. Given characteristic clinical presentations and good responsiveness to therapies targeting PCs, we suggested variant POEMS syndrome with undetectable M-protein.

Monoclonal PC dyscrasia is not only one of the two mandatory diagnostic criteria for POEMS syndrome [3]but also the crucial driving factor for pathogenesis [1, 2, 6, 7]. M-protein is an abnormal immunoglobulin (Ig) produced by the malignant PCs or B lymphocytes, existing as Ig or Ig fragments. Based on the most widely recognized viewpoint, deposition of M-protein or related antigen (Ag)-antibody (Ab) reaction is the leading cause of peripheral neuropathy [7, 9, 10]. Although M-protein plays a vital role in the pathogenesis of POEMS syndrome, there have been reports of variant POEMS syndrome with negative M-protein. In 2017, Omar Al-Mayoof et al. reported a case of POEMS syndrome with unexplained peripheral neuropathy accompanied by exudative ascites. However, the M-protein wasn’t detected by electrophoresis [10]. Additionally, Jian Li et al. also reported 13 cases of POEMS syndrome variants without M-protein [11].

M-protein of only about 60–80% of patients with POEMS syndrome can be detected, most of which are IgG or IgA and most of the light chain is λ, rarely κ [1, 2]. The weak transmembrane permeability or decreased release of M-protein may cause negative results [12]. In addition, Scholars believe that PCs wouldn’t secrete M-protein at all the same as the non-secreted MM in a subset of POEMS syndrome [13, 14]. Compared with MM, POEMS syndrome has a low level of M-protein [9]. Limitation of measurements may fail to identify trace amounts of M-protein. The detection limit of PEP for M-protein is 0.30–0.70 g/dL, and that of IFE is about 0.10 g/dL [15]. FLC-R was introduced with significantly improved sensitivity [16]. When FLC-R combines with both PEP and IFE, the detection rate of PC neoplasms including MM, smoldering MM, Waldenström macroglobulinemia, and Light chain amyloidosis can reach 99-100% [16, 17]. However, 13-18% of patients with POEMS syndrome still have normal FLC-R, which adds to the difficulty of diagnosing POEMS syndrome [18]. Considering the fact that the M-protein is undetectable in some patients suffering from POEMS syndrome, what can be said about the monoclonal PCs of these individuals? In 2020, Li et al. reported 42 patients with newly diagnosed POEMS syndrome, whose median percentage of BM PCs was only 1.5% (0–10%) [19]. In a multicenter analysis of 108 cases with POEMS syndrome, Jurczyszyn et al. reported that through BM smears, 33% of patients had more than 10% PCs, 62% had less than 10% PCs, 5% of them had no PC [20]. Flow cytometry can be used to distinguish normal and abnormal PCs. Although flow cytometry gradually becomes more and more important in the diagnosis of PC neoplasms, the detection rate of monoclonal PCs is only 50-60% [21]. Samples with repeatedly diluted, severe hemolysis and coagulation, incorrect human operation factors, etc. may cause false negative results [22]. Therefore, some researchers have sought to use more sensitive detection methods to find evidence of monoclonal PC dyscrasia. Mass Spectrometry has significant advantages of fast and accurate analysis of M-protein, but its high price, poor results of reproducibility, and complex data processing make its application in clinical practice face many challenges [23]. Although flow cytometry based on the Euroflow standardized system has the advantages of high speed, accuracy, and high throughput, the standardized system hasn’t been established in China, so it also hasn’t been popularized in clinical practice [24]. For this case, we conducted the pathological biopsy of BM and diseased bones, which both showed negative results. It’s worth further investigation that the immunohistochemical results of diseased bones revealed CD163^+^CD68^+^Ki-67^+^ cells, which may represent macrophages or monocytes [25]. Currently, there is no data to show that these cells are involved in sclerotic bone lesions of POEMS syndrome [8]. In view that we merely punctured the posterior superior iliac spine, we can’t rule out the possibility of discovering monoclonal PCs in other bone lesions due to their multifocal distribution [26]. Domestic reports have indicated that the positive rate of BM biopsy is about 50% [11]. Linda N. Dao et al. reported that clonal PCs were detected by BM biopsy in 44/67 (65%) patients with POEMS syndrome [27]. So many researchers believe that we should repeatedly conduct BM biopsy, preferably at places where X-ray and radionuclide scanning imply bone lesions [28]. Additionally, the positive rate of diseased bone biopsy is high in diagnosing bone tumors, but there are few reports about its application in POEMS syndrome. Except for the limitations of the piercing site, puncture techniques and the experience of doctors can markedly affect the results of biopsy. Therefore, other methods, such as multi-point biopsy, incision biopsy, and multidisciplinary collaboration, should be adopted to reduce missed diagnoses and misdiagnoses. Because of the limitations of existing detection methods, negative results in M-protein and monoclonal PCs aren’t enough to reject the diagnosis of POEMS syndrome.

POEMS syndrome is the consequence of PC disorder. Hence, treatment objectives are eradicating dysfunctional PCs and M-protein and ultimately attaining complete hematologic remission, closely related to progressive free survival (PFS) and OS [5]. Anti-PC therapy has an irreplaceable position in the treatment of POEMS syndrome. For those with systemic disease, the top picks are demonstrated with induction chemotherapy followed up with high-dose melphalan and stem cell rescue if eligible. As for transplant-ineligible patients, lenalidomide combined with dexamethasone remains a preferred treatment option. Additionally, CD38 monoclonal antibodies, B cell maturation antigen-Chimeric antigen receptor T cells (BCMA-CART), and other therapies have also appeared in clinical research. Several studies have revealed that anti-VEGF monoclonal antibody has modest effects on patients with POEMS syndrome [29].

## Conclusion

Monoclonal PC dyscrasia (M-protein) while being a mandatory criterion for POEMS syndrome is undetectable in a considerable amount of patients that otherwise demonstrate typical symptoms. Here, we reported a case of variant POEMS syndrome with featured clinical manifestations, elevated VEGF levels, and good response to therapies targeting PCs but no evidence of M-protein. Therefore, negative results in M-protein and monoclonal PCs aren’t enough to reject the diagnosis of POEMS syndrome. It is imperative to recognize the variant form of POEMS syndrome.

## Data Availability

The data and materials that support the findings of this article are available from the corresponding author upon reasonable request.

## Abbreviations

PC: Plasma cell
VEGF: Vascular endothelial growth factor
PEP: Protein electrophoresis
IFE: Immunofixation electrophoresis
FLC: R-Ratio of free light chains
IL: 6-Interleukin-6
IL: 1β-Interleukin-1β
TNF: α-Tumor necrosis factor-α
PET: CT-Positron emission tomography-computed tomography
MMT: Manual muscle test
PRL: Prolactin
FSH: Follicular estrogen
E2: Estradiol
P: Progestational hormone
T: Testosterone
COR: Cortisol
MDP: Methylene-diphosphonate
SPECT/CT: Single Photon Emission Computed Tomography/Computed Tomography
BM: Bone marrow
MM: Multiple myeloma
CD: Castleman Disease
MCD: Multicentric Castleman Disease
OS: Overall survival
GBS: Guillain Barre Syndrome
CIDP: Chronic inflammatory demyelinating polyradiculoneuropathy
Ig: Immunoglobulin
Ag: Antigen
Ab: Antibody
PFS: Progressive free survival
BCMA: CART-B cell maturation antigen-Chimeric antigen receptor T cells
